# Delivering the promises of trait‐based approaches to the needs of demographic approaches, and *vice versa*

**DOI:** 10.1111/1365-2435.13148

**Published:** 2018-06-17

**Authors:** Roberto Salguero‐Gómez, Cyrille Violle, Olivier Gimenez, Dylan Childs

**Affiliations:** ^1^ Department of Zoology University of Oxford Oxford UK; ^2^ Evolutionary Biodemography Laboratory Max Planck Institute for Demographic Research Rostock Germany; ^3^ Centre for Biodiversity and Conservation Science University of Queensland St Lucia Qld Australia; ^4^ CEFE, CNRS Univ Montpellier Univ Paul Valéry Montpellier 3, EPHE, IRD Montpellier France; ^5^ Department of Animal & Plant Sciences The University of Sheffield Sheffield UK

**Keywords:** fast–slow continuum, fitness, functional trait, leaf economics spectrum, life‐history trait, macroecology, selection gradient, vital rate

## Abstract

1. Few facets of biology vary more than functional traits and life‐history traits. To explore this vast variation, functional ecologists and population ecologists have developed independent approaches that identify the mechanisms behind and consequences of trait variation.
2. Collaborative research between researchers using trait‐based and demographic approaches remains scarce. We argue that this is a missed opportunity, as the strengths of both approaches could help boost the research agendas of functional ecology and population ecology.
3. This special feature, which spans three journals of the British Ecological Society due to its interdisciplinary nature, showcases state‐of‐the‐art research applying trait‐based and demographic approaches to examine relationships between organismal function, life history strategies and population performance across multiple kingdoms. Examples include the exploration of how functional trait × environment interactions affect vital rates and thus explain population trends and species occurrence; the coordination of seed traits and dispersal ability with the pace of life in plants; the incorporation of functional traits in dynamic energy budget models; or the discovery of linkages between microbial functional traits and the fast–slow continuum.
4. Despite their historical isolation, collaborative work between functional ecologists and population ecologists could unlock novel research pathways. We call for an integrative research agenda to evaluate which and when traits are functional, as well as their ability to describe and predict life history strategies and population dynamics. We highlight promising, complementary research avenues to overcome current limitations. These include a more explicit linkage of selection gradients in the context of functional trait–vital rate relationships, and the implementation of standardised protocols to track changes in traits and vital rates over time at the same location and individuals, thus allowing for the explicit incorporation of trade‐offs in analyses of covariation of functional traits and life‐history traits.

## Sumario

1. Pocos aspectos varían más en biología que los caracteres funcionales y de historia vital. Para explorar esta vasta variación, los ecólogos funcionales y de poblaciones han desarrollado independientemente métodos que identifican los mecanismos y las consecuencias de dicha variabilidad.
2. Las colaboraciones entre investigadores que utilizan métodos basados en caracteres funcionales y de historia vital son bastante limitadas a día de hoy. Aquí argumentamos que éllo conlleva una gran oportunidad aún por explotar, ya que las fortalezas de ambas metodologías podrían revolucionar las agendas investigadoras de la ecología funcional y de poblaciones.
3. Este número especial, el cual incorpora tres de las revistas de la Sociedad Ecológica Británica debido a su carácter interdisciplinar, contiene investigaciones punteras en la aplicación de metodologías de caracteres funcionales y métodos demográficos para examinar relaciones entre funciones del organismo, estrategias de historia vital, y el rendimiento poblacional en varios reinos. Algunos ejemplos incluyen la exploración de cómo las interacciones de carácter funcional~ambiente afectan las tasas vitales para así explicar tendencias demográficas y ocurrencia de especies; la coordinación de algunas características de las semillas, su habilidad dispersora, y el ritmo vital en el reino vegetal; la incorporación de caracteres funcionales en modelos de presupuesto dinámico de energía; o el descubrimiento de enlaces entre caracteres funcionales en microbios y el continuo rápido‐lento.
4. A pesar del aislamiento histórico, las colaboraciones entre la ecología funcional y poblacional podrían abrir novedosas rutas de investigación. Hacemos una llamada para el desarrollo de una agenda investigadora integradora que evalúe cuáles y cuándo los caracteres son funcionales, así como su habilidad para predecir estrategias de historia vital y dinámicas poblacionales. Asimismo, resaltamos aproximaciones investigadoras complementarias y prometedoras para superar las limitaciones actuales, incluyendo una vinculación más explícita de los gradientes de selección en las relaciones carácter funcional~tasa vital, o protocolos estandarizados para examinar cambios temporales en caracteres funcionales y tasas vitales en la misma población e individuos. Éllo permitirá incorporar explícitamente los compromisos energéticos en los análisis de co‐variación de caracteres funcionales y de historia vital.

## Résumé

1. Peu de facettes de la biologie varient autant que les traits fonctionnels et les traits d'histoire de vie. Pour explorer cette grande variation, les écologues fonctionnels et les écologues des populations ont développé des approches indépendantes pour identifier les mécanismes sous‐jacents et les conséquences des variations de ces traits.
2. Les interactions entre les chercheurs utilisant les approches soit basées sur les traits fonctionnels soit sur la démographie sont rares. Il s'agit sans aucun doute d'une opportunité manquée car les forces combinées des deux approches pourraient accélérer les agendas de recherches aussi bien de l'écologie fonctionnelle que de l'écologie des populations.
3. Ce numéro spécial, à cheval sur trois journaux de la Société Britannique d'Ecologie, illustre les dernières recherches appliquant les approches qui s'appuient sur les traits et la démographie pour examiner les relations entre les fonctions des organismes, les stratégies d'histoire de vie, et les performances des populations pour différents groupes taxonomiques. Les exemples incluent: (i) l'exploration de la façon dont les interactions traits fonctionnels x environnement affectent les taux vitaux et expliquent l'occurrence des espèces et les tendances populationnelles; (ii) la façon dont le rythme de vie des plantes covarie avec les traits des graines et la capacité de dispersion de celles‐ci; (iii) l'incorporation des traits fonctionnels dans les modèles de budget énergétique; (iv) ou encore la découverte d'un lien entre les traits fonctionnels des microbes et le gradient lent‐rapide.
4. Malgré leur isolement historique, le travail collaboratif entre les écologues fonctionnels et les écologues des populations pourrait ouvrir de nouvelles voies de recherche. Nous appelons ainsi à une recherche intégrative pour évaluer quels traits sont fonctionnels, et dans quelles mesures, ainsi que leur capacité à décrire et prédire les stratégies d'histoire de vie et la dynamique des populations. Nous mettons en lumière des pistes de recherche prometteuses et complémentaires pour dépasser les limites actuelles. Ces pistes incluent une liaison plus explicite des gradients de sélection dans le contexte de la relation trait fonctionnel~taux vitaux et l'implémentation de protocoles standardisés pour suivre les variations temporelles des traits fonctionnels et les taux vitaux en un même lieu et sur les mêmes individus, permettant ainsi la prise en compte explicite des compromis dans l'analyse de la covariation entre les traits fonctionnels et les traits d'histoire de vie.

## 1 INTRODUCTION

**Figure 1:**
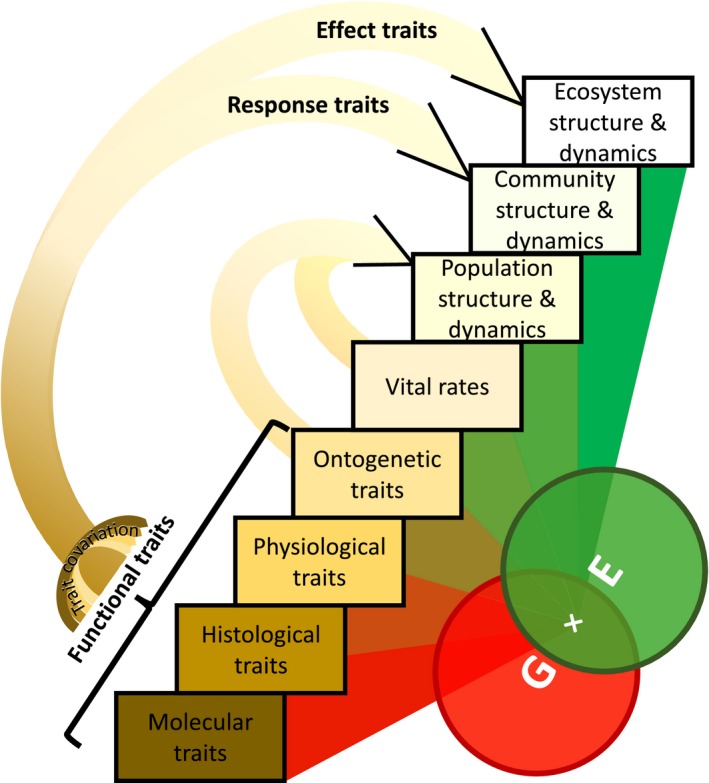
Two of the most widely used approaches to examine drivers and consequences in trait variation include the trait‐based approach and the demographic approach. In the trait‐based approach, molecular (e.g. oxidative stress), histological (e.g. bone/wood density), physiological (e.g. photosynthetic rate) and ontogenetic traits (e.g. adult height) are used to explore trait–trait covariation (e.g. Chave et al., 2009; Díaz et al., 2016; Wright et al., 2004). These so‐called *functional traits* are also used to upscale to describe the structure and dynamics of communities (e.g. McGill et al., 2006) and ecosystems (e.g. Gross et al., 2017) using response traits and effect traits. In this up‐scaling, typically the demographic compartment (vital rates and populations) is not considered. The demographic approach examines how *vital rates* (e.g. survival, development, reproduction) scale with ontogenetic characteristics of individuals (e.g. age, size, development) to inform on population structure and dynamics (e.g. Caswell, 2001). Both trait‐based and demographic approaches share similarities in the questions they target (e.g. ***G***enetics × ***E***nvironment interactions; Barks, Dempsey, Burg, & Laird, 2018; Vasseur et al., 2018), and the recent macroecological patterns of trait covariation they have reported (Díaz et al., 2016; Salguero‐Gómez, Jones, Archer, et al., 2016; Salguero‐Gómez, Jones, Jongejans, et al., 2016). In this editorial, we argue that research using both approaches can advance the research agenda of functional ecology and population ecology (See Section 2)

The interest in understanding the causes and consequences of the considerable variation in organismal traits has fuelled decades of ecological studies (Bolnick et al., 2011; Brown, 1995; Calow, 1987; Sutherland et al., 2012). This research agenda has benefitted from numerous approaches that (a) classify and compare organismal characteristics, and (b) explore the mechanisms behind such a variation as well as its ecological, evolutionary and conservation implications. Two of the most prominent advances in addressing these goals are the trait‐based approach and the demographic approach (Figure 1).

Trait‐based approaches focus primarily on the linkage between molecular/histological/physiological/ontogenetic/behavioural attributes, their functions and the impact that the environment has on them (Calow, 1987; Garnier, Navas, & Grigulis, 2015; Keddy, 1992). The common currency of this approach is the *functional trait*,* “a surrogate of organismal performance … which impacts fitness indirectly via their effects on growth, reproduction and survival”* (Violle et al., 2007). In recent decades, this approach has also been expanded to understand and predict the structure, dynamics, and functioning of communities and ecosystems (Díaz & Cabido, 1997; Funk et al., 2017; Lavorel, 2012; Lavorel & Garnier, 2002; McGill, Enquist, Weiher, & Westoby, 2006; Violle, Reich, Pacala, Enquist, & Kattge, 2014). *Response traits* reflect how organisms respond to their environment, with implications for community structure and dynamics, whereas those that directly influence ecosystem properties are known as *effect traits* (*sensu* Lavorel & Garnier, 2002; Figure 1).

The linkage between traits and fitness has often been regarded as nonessential for addressing questions that are central to functional ecology. For instance, trait‐based approaches have been advocated to predict species abundance (Shipley, Vile, & Garnier, 2006; Shipley et al., 2016), ecophysiological trade‐offs (e.g. Chave et al., 2009; Díaz et al., 2016; Wright et al., 2004) and ecosystem‐level processes (Garnier et al., 2015), all without considering demographic variation. Nonetheless, other authors consider the link between functional traits and fitness fundamental to carry out up‐scaling research that allows linking traits to communities and/or ecosystems (Enquist et al., 2015). Some authors are starting to use demography to address traditional questions in functional ecology, including the ultimate causes of ecophysiological relationships (Donovan, Maherali, Caruso, Huber, & de Kroon, 2011; Vasseur et al., 2018); the identification of species coexistence mechanisms (Adler, Fajardo, Kleinhesselink, & Kraft, 2013; Laughlin, Strahan, Adler, & Moore, 2018; McGill et al., 2006); or the up‐scaling to ecosystem processes (e.g. Trait‐Driver Theory; Enquist et al., 2015).

Demographic approaches are used in ecology to understand and predict fitness variation, population dynamics and population structure. Population ecologists typically operate at the individual and population levels (Harper, 1964; but see Hart & Keough, 2009; Silvertown, Franco, & McConway, 1992). *Vital rates* (Caswell, 2001) and their emergent *life‐history traits* and *life history strategies* (Stearns, 1982) constitute the common currencies of the demographic approach. Vital rates quantify an individual's investment into its own maintenance (i.e. survival), development, and into the next generation (i.e. reproduction). Together, vital rates combinations determine not only an individual's fitness (Lande, 1982; Roff, 2002) but also its key life‐history traits, such as the rate of senescence (Jones et al., 2014), generation time (Gaillard et al., 2005) or degree of iteroparity (Hughes, 2017). The combination of an organism's life‐history traits results in its life history strategy, such as being a long‐lived masting species (Bogdziewicz, Steele, Marino, & Crone, 2018), or a monocarpic perennial species (Hughes, 2017).

Demographic approaches examine vital rates with an explicit recognition of the importance of individual differences in those rates (Gimenez, Cam, & Gaillard, 2017; Tuljapurkar & Caswell, 1997; Vindenes, Engen, & Saether, 2008). Contributions of individuals to population dynamics typically differ as a function of underlying ontogenetic traits (Figure 1) such as age, development, or size. The explicit incorporation of among‐individual heterogeneity makes the demographic approach fundamentally different to most trait‐based approaches. Demography builds on among‐individual variation as the starting point, and then often attempts to scale up to population processes, whereas trait‐based approaches often (but not always; see, e.g. Albert, Grassein, Schurr, Vieilledent, & Violle, 2011; Violle et al., 2012) start with species‐level averages of functional trait values. Nonetheless, ontogenetic traits (e.g. juvenile vs. adult, age, size at maturity) only depict phenomena, to a large extent, rather than underlying mechanisms (Salguero‐Gómez, 2017). The treatment of individual heterogeneity in population ecology from more basal levels of functional traits (e.g. molecular, histological, physiological; Figure 1) remains largely unexplored.

### 1.1 Motivation and goals

Though fundamentally different in approach, recent independent efforts using trait‐based or demographic approaches have resulted in the recognition of similar global patterns of trait covariation. From a trait‐based perspective, Díaz et al. (2016) reported two main axes of variation for vascular plants, with one representing leaf investment‐revenue trade‐offs (i.e. the Leaf Economics Spectrum; Wright et al., 2004), and a second axis running along organismal/organ size. From a demographic perspective, research on mammals and birds (Bielby et al., 2007; Gaillard et al., 1989; Sæther, 1987), reptiles (Dunham & Miles, 1985) and plants (Salguero‐Gómez, Jones, Jongejans, et al., 2016) has found two independent axes of life‐history trait covariation. Here, the dominant axis classifies life according to the pace of life of organisms, which is associated with organismal size (Gaillard et al., 2005). The secondary axis classifies life according to how organisms reallocate resources from maintenance into reproduction.

The prospect that these and other recent macroecological studies (e.g. Rüger et al., 2018) might have just scratched the surface of functional trait–vital rate relationships has motivated this special feature. Our motivation is further fuelled by the possibility that the promise of both approaches can greatly boost progress in functional ecology and population ecology (Section 2). The primary goal of this special feature is to provide a broad perspective on the state‐of‐the‐art research using both trait‐based and demographic approaches. Here, we (a) introduce the rich, vibrant research agenda at the interface of the exploration of functional traits and vital rates, (b) document how the exploration of functional traits and demographic processes can benefit ecological, evolutionary and conservation research, and (c) suggest future directions. Ultimately, we hope that the collection of articles in this special feature will encourage novel, universal theories and experimental approaches to explain how organismal function, life‐history traits and strategies, and population performance are interrelated.

## 2 BRIDGING TRAIT‐BASED AND DEMOGRAPHIC QUESTIONS AND APPROACHES

There has been a historical lack of research at the interface of functional ecology and population ecology (Salguero‐Gómez, 2017; Shipley et al., 2016). In this section, we highlight how the goals of each discipline can be met with the promises and strengths of the other via complementary approaches and theories.

### 2.1 How can demographic approaches help functional ecology?

Research using trait‐based approaches has mostly developed along three axes: first, analysing interspecific trait covariation to explore general principles and laws that constrain global phenotypic diversity; second, understanding and predicting responses of organisms, communities, and ecosystems to environmental changes; finally, quantifying the effect of biodiversity on ecosystem processes and services. Below we argue how these three research axes could benefit from demographic approaches.

Functional trait‐based ecology has searched for generalisation in the phenotypic diversification of life. These efforts have repeatedly reported patterns in macroevolution thought to reflect universal laws (Grime & Pierce, 2012). In plants, one of these general patterns is the Leaf Economics Spectrum (Wright et al., 2004), whereby species are ranked along a continuum driven by both biophysical and ecophysiological constraints and natural selection. At one end, organisms are characterised by a high investment in metabolism, while at the other, by slow metabolism and a large investment in protection against abiotic and biotic pressures. The Leaf Economics Spectrum mirrors the fast–slow continuum (Reich, 2014; Salguero‐Gómez, 2017), developed within the realm of Life History Theory (Stearns, 1976). However, empirical demonstrations of the adaptive nature of the trade‐offs that modulate the spectrum of leaves remain relatively scarce (Moles, 2018). Exploring how and why functional traits covary intraspecifically is certainly possible using genetics (Donovan et al., 2011; Vasseur et al., 2018), but this approach is impractical at large spatial or taxonomic scales due to the costs involved. Population ecology, which has nurtured comparative analysis since its inception (Gaillard et al., 1989; Harper, 1977; Sarukhán & Harper, 1973), represents a promising avenue to explore the fitness variation underlying such cross‐species “universal” laws.

Identifying the response traits that reflect the adaptation of organisms to their environment is a mainstream approach in functional ecology. A pivotal step in such trait‐based approaches is to build interspecific trait~environment relationships. However, these connections are poorly understood in most taxa (Violle et al., 2014). Further, such correlative exercises remain a weak demonstration of the adaptive nature of functional traits and their role in adaptation (Moles, 2018). The reasons for this weakness include the static nature of the approach, which impedes tracking organismal responses over time, and trait covariation, which can blur causal trait~environment relationships (Wüest, Münkemüller, Lavergne, Pollock, & Thuiller, 2017). In contrast, the sheer volume of demographic data and approaches (below) makes population ecology an ideal ally of functional ecology to overcome this limitation.

Using functional traits to explain species coexistence has been a popular trait‐based research avenue in the last decade (e.g., Kraft, Godoy, & Levine, 2015; Kraft, Valencia, & Ackerly, 2008). This approach is based on the strong assumption that trait variation reflects species' niche breath (Kraft et al., 2015; Violle & Jiang, 2009). While conceptually appealing (McGill et al., 2006), trait‐based community ecology studies have often used traits selected without *a priori* examination of their role in fitness nor in assembly processes. In addition, there has been growing recognition of the role of intraspecific trait variability in community assembly (Jung, Violle, Mondy, Hoffmann, & Muller, 2010; Violle et al., 2012). However, the assessment of such variability remains largely phenomenological (Taudiere & Violle, 2015). Evaluating the effect of ontogeny, as typically carried out in population ecology (Caswell, 2001; Ebert, 1999), on community‐level trait distribution and the maintenance of species coexistence would fill a major theoretical gap in community ecology. Likewise, a demographic approach could be used to model community dynamics, including trait‐based assembly rules (Adler, Ellner, & Levine, 2010; Teller, Adler, Edwards, Hooker, & Ellner, 2016).

Several studies have demonstrated the utility of trait‐based approaches to upscale from the functioning of organisms to the functioning of ecosystems. For example, leaf traits have been used to relate the instantaneous functioning of organs (e.g. photosynthesis) to organisms (e.g. plant relative growth rate) and ecosystems (e.g. primary productivity) (Garnier et al., 2004, 2015; Violle et al., 2007). In such cases, simple integrative functions (Violle et al., 2007), like the calculation of community‐mean trait values, have proven useful. The distribution of traits within an ecosystem is also expected to reflect the complex mechanisms underlying ecosystem processes (Gross et al., 2017; Ricotta & Moretti, 2011). Enquist et al. (2015) developed the Trait‐Driver Theory to link the environment and the distribution of fitness‐related traits to ecosystem functioning. Integrating fitness variation over the life cycle will require concepts and methods from population ecology. Population ecology can also be useful for the assessment of long‐term ecosystem functioning (Kuebbing et al., 2018) because the dynamics and stability of ecosystems cannot be reliably tracked using trait‐based snapshot approaches (Enquist et al., 2015).

### 2.2 How can trait‐based approaches help population ecology?

The operational unit of population ecology is the individual within its population (Harper, 1964). However, due to convenience, population ecologists often group individuals into classes and then estimate the class‐specific vital rates (Tuljapurkar & Caswell, 1997). In animal population ecology, the most widely used state variable has historically been age (Caswell, 2001; Ebert, 1999), while in plant population ecology, it is size (Caswell, 2001; Gibson, 2014; Salguero‐Gómez et al., 2015). However, vital rate variation associated with age and size ultimately reflects differences in functional traits (Figure 1) such as specific leaf area, wood density or metabolic rate (Adler, Fajardo, et al., 2013; Adler, Salguero‐Gómez, et al., 2013; Kurta & Ferkin, 1991; Visser et al., 2016), or even physiological trait networks (Cohen, Martin, Wingfield, McWilliams, & Dunne, 2012).

A trait‐based approach could improve the predictive capacity of population ecology. This is important and urgent due to the linkages between demography and conservation science: whether a species' population goes locally extinct or becomes invasive is determined to a large extent by its vital rates (Morris & Doak, 2002; Silvertown, Franco, & Menges, 1996). The limitation of the demographic approach, however, is that conservation science often requires robust, informed recommendations on time‐scales that are incompatible with the collection of demographic data (Conde, Flesness, Colchero, Jones, & Scheuerlein, 2011). Demography is a data‐hungry discipline (Griffith, Salguero‐Gómez, Merow, & McMahon, 2016), where studies with hundreds of individuals spanning four or more years are not uncommon (Salguero‐Gómez et al., 2015; Salguero‐Gómez, Jones, Archer, et al., 2016; Salguero‐Gómez, Jones, Jongejans, et al., 2016). One of the promises of the trait‐based approach, to characterise a system's structure and dynamics with a single or few visits to the field (Violle et al., 2007), may greatly aid population ecology.

Perturbation analyses are widely used to address fundamental questions in ecology (Heppell, Pfister, & de Kroon, 2000; Silvertown et al., 1992), evolution (Caswell & Salguero‐Gómez, 2013) and conservation biology (Silvertown et al., 1996). First introduced to ecology by de Kroon, Plaisier, van Groenendael, and Caswell (1986), sensitivity and elasticities quantify the absolute and the relative effect, respectively, of a small change in a demographic process on a descriptor of the population. The descriptor of choice is typically the long‐term, deterministic population growth rate, λ, which is a proxy for individual fitness averaged across the population (Caswell, 2001). The sensitivity of population growth rate to a vital rate represents a selection gradient (*sensu* Lande, 1982): the partial derivative of fitness with respect to a small change in a trait value (van Tienderen, 2000). The examination of the relationship between functional traits and vital rates in a full‐life cycle context can be used to quantify the sensitivity of fitness to functional trait variation and identify the pathways through which its effects play out (e.g. Adler, Fajardo,et al., 2013; Adler, Salguero‐Gómez, et al., 2013).

Lastly, population ecology has long been instrumental for conservation ecology through the evaluation of populations at risk of extinction (Morris & Doak, 2002). Interestingly, the target of conservation has been questioned through many angles (Beissinger, 2015). In particular, the rarity of functions (i.e. functional rarity) can be as important to protect as the rarity of species (Violle et al., 2017). This research area is in its infancy, and it appears urgent to evaluate the link between demography and functional traits (identity and values) to evaluate the relevance of conserving rare traits and functions in an ecosystem.

## 3 NOVEL CONTRIBUTIONS OF THIS SPECIAL ISSUE

This special feature includes 11 articles that combine cutting‐edge trait‐based and demographic to address timely ecological and evolutionary questions. The taxonomic and methodological breath of their research is shown by the fact that the special feature spans three of the British Ecological Society's journals: *Functional Ecology*,* Journal of Animal Ecology* and *Journal of Ecology*. The key innovations of these contributions are presented here in four main themes:

### 3.1 Theme 1: Microhabitat modulates function~performance relationships

The relationships between traits and demographic performance are typically modulated by the environment (Ellsworth & Reich, 1992). This specificity is likely caused by genetic differences and phenotypic plasticity of both functional traits (Messier, McGill, & Lechowicz, 2010) and vital rates (Coutts, Salguero‐Gómez, Csergő, & Buckley, 2016; Figure 1). This may explain why recent global analyses linking traits and vital rates at different sites have lacked high predictive ability (Adler, Fajardo,et al., 2013; Adler, Salguero‐Gómez, et al., 2013; Salguero‐Gómez, 2017). To overcome this challenge, one must obtain high‐resolution, individual‐level information about functional traits, vital rates, and the local environment. Blonder et al. (2018) use vital rate data from a community of alpine plant species, for which above‐ and below‐ground functional traits and microenvironmental conditions were measured at the individual level. The predictive models of community dynamics perform much better once microhabitat conditions are considered. Importantly, they also show that vital rates key to the presence and distribution of most species can be predicted by functional traits. Leibman, Rowe, Koski, and Galloway (2018) evaluate how plasticity in floral traits interacts with the environment to shape fitness via the degree of outcrossing. The authors use several populations of *Campanula america* to test how pollen limitation depends on pollinator visitation rates, and how floral traits that are related to selfing respond to pollinator availability. They find that populations with a high selfing potential have a greater degree of trait plasticity to pollinator presence.

### 3.2 Theme 2: The functionality of traits is fitness‐component specific

The ability of traits to predict fitness components remains largely unexplored (Salguero‐Gómez, 2017; Shipley et al., 2016). This is a glaringly missing step in trait‐based approaches, given that functional traits are typically defined as organismal features that *impact on fitness* (Violle et al., 2007). This special feature provides key contributions that explore trait~fitness relationships. Garnier et al. (2018) link nine functional traits with the vital rates of 53 plant species in a 28‐year study where management was intensified. Theirs is one of the few studies to date to have demonstrated how changes in traits affect demographic change (but see Flores, Hérault, Delcamp, Garnier, & Gourlet‐Fleury, 2014). Garnier and collaborators find that species that increased their abundance in response to intensified management were short‐lived and had high leaf phosphorus and low leaf dry matter content. Wenk, Abramowicz, Westoby, and Falster (2018) test the terminal investment hypothesis, whereby resource allocation should be fully diverted to reproduction towards the end of an organism's life in order to maximise its fitness. The authors' explicit incorporation of the plant's investment into maintenance vs. expansion of above‐ground size is the key to reconciling theory (Kozlowski, 1992) and previous contradicting evidence (e.g. Wenk & Falster, 2015). Using a combination of trait‐based and demographic approaches on age‐known individuals of 14 Australian woody species, the authors find that most of these species allocated almost all of their resources to reproduction at advanced ages. Cheap‐leaf producing species peak faster in reproductive allocation, whereas lower‐than‐average reproductive allocation is associated with greater height, an ontogenetic trait associated with the fast–slow continuum (Salguero‐Gómez, Jones, Jongejans, et al., 2016).

### 3.3 Theme 3: Coordination of traits into syndromes

The coordination of functional traits and life history strategies is critical in understanding why traits vary and their consequences. Several contributions in this special feature develop integrative frameworks to that end. Beckman, Bullock, and Salguero‐Gómez (2018) focus on a phenomenon that has not typically been viewed as a functional trait: dispersal. They bring together global data on key anatomical traits, life‐history traits, and dispersal ability and mode of dispersal for 141 plant species to evaluate emerging dispersal syndromes (*sensu* Ronce & Clobert, 2012). They report a novel axis, independent of the fast–slow continuum and reproductive strategies axis, that classifies species according to their reproductive output and degree of iteroparity (Salguero‐Gómez, Jones, Jongejans, et al., 2016). Along this axis, species with a high lifetime reproductive success, extended reproductive windows, high senescence rates and low propensity towards shrinkage disperse seeds further. Ellers et al. (2018) expand the definition of functional traits to include degree of tolerance to abiotic stress conditions. They analyse changes in multidimensional trait distribution (morphological, physiological, behavioural and life‐history traits) in dominant groups of soil fauna key for ecosystem services. The authors test whether the vertical distribution of species in the soil profile correlates with trait variation based on trait richness, evenness and divergence, and find three axes of variation that structure soil invertebrate traits. The dominant axis is aligned with soil depth and has lower trait diversity at the surface. Marshall, Petterson, and Cameron (2018) evaluate the causes and consequences of offspring size across hundreds of plants and animals. Offspring size is a good descriptor of a classical trade‐off that individuals face: invest in one‐self vs. the next generation. The authors find that offspring size is positively correlated with latitude in fish, amphibians, invertebrates and birds, but negatively correlated in plants and turtles. They allude to the developmental window hypothesis, whereby species that produce large offspring need more time to achieve the same ontogenetic trait value than species that produce small offspring. Finally, Ghedini, White, and Marshall (2018) examined how individual metabolic rates scale up to the community level. Through a series of chronosequences of sessile marine invertebrate communities, the authors determine whole‐community metabolic scaling across successional stages. They then examine whether the sum of the individual metabolic rates for the dominant species predict the overall community metabolic rate. Contrary to their initial predictions, community metabolism scaled isometrically with community biomass along the succession. The explicit incorporation of the population structure greatly improved their model's community‐level predictions.

### 3.4 Theme 4: Integration of functional traits into demographic approaches

Demographic models calculate important metrics of population performance, life‐history traits and selection gradients (Ellner, Childs, & Rees, 2016; Metcalf, McMahon, Salguero‐Gómez, & Jongejans, 2013; Morris & Doak, 2002). However, the state variables typically used in these models (e.g. size, developmental stage, age) are considered too phenomenological to explore underlying mechanisms of variation (Salguero‐Gómez, 2017). Jenouvrier et al. (2018) develop a hierarchical matrix population model integrating functional traits and vital rates to predict population responses of the black‐browed albatross (*Thalassarce melanophris*) to changes in climate. Their model allows the authors to evaluate the relative contributions to population dynamics of trait shifts and direct climate effects. The authors find significant interactions between early life cycle stages, increases in sea surface temperature and multiple functional traits, as well as cross‐seasonal carry‐over effects on population growth rate. Smallegange and Ens (2018) investigate the predictive performance of a mechanistic, trait‐based demographic model. They construct an integral projection model (IPM) for which the vital rates of survival, growth and reproduction are informed by a dynamic energy budget (DEB; Kooijman & Troost, 2007; van der Meer, 2006). They then investigate the capacity of their model to predict the dynamics a laboratory microcosm. In contrast to the classical phenomenological perspective via ontogenetic traits (e.g. size; Figure 1), the integration of energy conservation principles into IPMs permits predictions under novel environmental conditions. For example, the authors examine the sensitivity of population responses to climate change with respect to key life‐history traits, such as maximum reproductive rate, and to functional traits like individual length at birth. Lemaître et al. (2018) examine the role of complex secondary sexual traits, deer antlers, on fitness. Through a combination of trait‐based and demographic approaches, they quantify the costs of investment in antlers onto fitness components late in life evoking theories of ageing (Shefferson, Jones, & Salguero‐Gómez, 2017) and life history (Stearns, 1976, 1982). Specifically, they evaluate the costs of producing large antlers early in life onto survival, body mass and antler size during adulthood in two European roe deer (*Capreolus capreolus*) populations. The authors find no delayed costs of sexual traits developed early in life on fitness; on the contrary fawns with the longest antlers also had the highest body mass as adults, suggesting that this trait is an honest signal.

## 4 UNIFYING QUESTIONS AND APPROACHES

Our call for more collaborative research using trait‐based and demographic approaches is not a new one (Martínez‐Garza, Bongers, & Poorter, 2013; Poorter & Bongers, 2006; Poorter et al., 2008; Silva et al., 2017; Yang, Cao, & Swenson, 2018). Although advances are being made in this direction, there remain considerable opportunities to further knowledge and scientific advance by integrating both approaches. This special feature was designed to galvanise progress in this area; its invited contributions represent a leap forward towards the fruitful marriage of questions and approaches in functional ecology and population ecology. However, some additional aspects require further attention to consummate this union.

### 4.1 Research using trait‐based and demographic approaches needs to examine the same individual

The macroecological patterns reported regarding investments on leaf (Wright et al., 2004), wood (Chave et al., 2009), root (Roumet et al., 2016) and organismal/organ size (Díaz et al., 2016) have become widely cited. However, the data they are based on often come from distant locations for a given species, do not typically consider environmental differences, and contain functional trait values that have not been measured on the same individual. This approach greatly limits our ability to evaluate whether and how the most prevalent tenet of ecology and evolution, the *trade‐off* (Roff, 2002; Stearns, 1992), and microhabitat conditions affect world‐wide patterns of trait covariation. Carrying out functional and demographic fieldwork on the same species' individuals, especially for multiple species, is extremely labour intensive and tedious—but see Blonder et al. (2018), Marshall et al. (2018) and Garnier et al. (2018). Luckily, new demographic approaches such as IPMs are robust to low sample sizes (Ramula, Rees, & Buckley, 2009), allowing us to improve species and spatial replication. Furthermore, the inverse problem of demography, whereby vital rates can be estimated from static population structure, can help prioritise field efforts (Evans, Merow, Record, McMahon, & Enquist, 2016; González, Martorell, & Bolker, 2016). The integration of functional trait collection via the StrateGo Network (Salguero‐Gómez, 2018) on populations where demographic data are already being collected world‐wide through the COMPADRE Plant Matrix Database (Salguero‐Gómez et al., 2015) is proving instrumental to address this limitation.

### 4.2 Integration of traits across the full anatomy of the organism

In the case of the Plant Kingdom, below‐ground processes remain widely unknown to functional ecology and population ecology. This is particularly surprising, as root traits play critical ecosystem service roles (Laliberté, 2017) and below‐ground plant biomass can account for over 50% of their total biomass (Eshel & Beeckman, 2013). Nonetheless, frameworks of functional trait variation focus mostly on above‐ground traits (Chave et al., 2009; Díaz et al., 2016; Wright et al., 2004). Similarly, studies describing plant population dynamics often ignore below‐ground dynamics (but see Pregitzer, Hendrick, & Fogel, 1993). Noninvasive technologies that quantify vital rates of below‐ground plant components, and the recent release of the Fine Root Ecology Database (Iversen et al., 2017) will help overcome this challenge.

### 4.3 No more correlative analyses without a priori hypotheses

In a recent publication, Moles (2018) made an eloquent call for more hypothesis‐driven research, following the recent impetus of global patterns in plant traits. Here, we reiterate this call in plant ecology and wish to extend it to other kingdoms. The availability of functional trait (e.g. Kattge et al., 2011; Klimešová, Danihelka, Chrtek, de Bello, & Herben, 2017; Knevel, Bekker, Bakker, & Kleyer, 2003; Kühn, Durka, & Klotz, 2004; Madin, Anderson, et al., 2016; Razafindratsima, Yacoby, & Park, 2018; Tamme et al., 2014; Wang et al., 2018), demographic (e.g. NERC Centre for Population Biology, 2003; Salguero‐Gómez et al., 2015; Salguero‐Gómez, Jones, Archer, et al., 2016; Salguero‐Gómez, Jones, Jongejans, et al., 2016; Santini, Isaac, & Ficetola, 2018) and life‐history trait data (e.g. Froese, 2014; Jones et al., 2009; Myhrvold et al., 2015; Strier et al., 2010) for thousands of species world‐wide, together with analytical packages (e.g. Blonder et al., 2017; Maitner et al., 2017; Taudiere & Violle, 2015), makes the task of quantifying and classifying variation in functional traits and vital rates more accessible than ever. However, researchers should be aware of the double‐edged sword presented by the big data~software tandem: A careful examination of the research questions *prior* to running “big‐data” analyses is vital in avoiding purely correlative, data‐mining exercises (Džeroski, 2008). Both functional ecology and population ecology have made great progress at classifying functions and life history strategies. What is needed now is to understand the causes and implications of the variation using the scientific method: Hypotheses come first, analyses second.

### 4.4 Does the holy grail of cross‐taxonomic trait exist?

In the last decades, plant functional ecologists have achieved a titanic progress towards the standardisation of functional trait data collection and its ecological interpretation for (predominantly) vascular plants (Cornelissen et al., 2003; Pérez‐Harguindeguy et al., 2013). Similar efforts have started to emerge across other taxonomic groups, including birds (e.g. Negret, 2016; Renner & van Hoesel, 2017), corals (Madin, Hoogenboom, et al., 2016), amphibians and freshwater fish (Negret, 2016), invertebrates (Bertelsmeier, 2017; Brousseau, Gravel, & Handa, 2018; Moretti et al., 2016), phytoplankton (Irwin & Finkel, 2017) or mycorrhizal fungi (Chagnon, Bradley, Maherali, & Klironomos, 2013). A commonality of most of these protocols, however, is their taxa specificity: Obviously wood density is not a good trait for animals, just as wing length is not for plants. While it is entirely possible that no single set of traits may explain function across the tree of life, we still deem this an important task to pursue for the maturation of ecology. This “holy grail” of functional trait sets would put trait‐based ecology at the same high level of macroecological predictability as population ecology, because in the case of population ecology, the vital rates (survival, development, reproduction) are universal to *any* organism. We call for more work evaluating variation and the predictive ability of metabolic rate for vital rates, as the rate‐of‐living hypothesis states that metabolic rate is inversely related to longevity (Rubner, 1908), and metabolic rate is a trait that links functional ecology and individual performance via the Metabolic Theory of Ecology (Brown, Gillooly, Allen, Savage, & West, 2004).

## 5 CONCLUSIONS

Functional traits have so far informed community (Lavorel & Garnier, 2002; McGill et al., 2006) and ecosystem structure, dynamics and functions (Díaz & Cabido, 1997; Gross et al., 2017) without requiring a mediating demographic layer (Figure 1). So why a call to integrate trait‐based and demographic approaches? The papers in this special feature build bridges between functional ecology and population ecology to understand core ecological and evolutionary questions such as which and how traits are filtered by the environment, or the selective pressures that shape them, and how they may change with time. It is remarkable that, despite the lack of communication, macroecological studies aimed at describing variation in functional traits and life‐history traits have found similar drivers of this variation (Díaz et al., 2016; Salguero‐Gómez, Jones, Jongejans, et al., 2016). Recently, both approaches have found a common ground through relationships between specific leaf area and generation time (Rüger et al., 2018; Salguero‐Gómez, 2017). We anticipate that the next decade will witness a fruitful marriage between trait‐based and demographic approaches, not only because the functionality of traits must be evaluated with respect to their effects on fitness components (Adler, Fajardo,et al., 2013; Adler, Salguero‐Gómez, et al., 2013; Lande, 1982), but also, perhaps most importantly, because many of the current needs of functional ecology and population ecology may be satiated with the theoretical frameworks and methodological strengths of the other.

## AUTHORS' CONTRIBUTIONS

R.S.‐G. and C.V. laid down the foundations to the special feature. R.S.‐G. oversaw and coordinated its development and integration. R.S.‐G. wrote the first version of the editorial and integrated feedback from coauthors and reviewers. C.V. contributed some portions regarding the state of the art in trait‐based ecology. All authors contributed to the article and gave final approval for publication.

## CONFLICT OF INTEREST

The authors have no conflict of interests to declare.

## DATA ACCESSIBILITY

No data were used in this article.
